# A Practical Treatise on Venereal Diseases

**Published:** 1838-01

**Authors:** 


BRITISH AND FOREIGN

MEDICAL REVIEW,

FOR JANUARY, 1838.

### PART FIRST.

### Analytical and Critical Reviews.

### Art. I.

1. *Traité pratique des Maladies Vénériennes, comprenant l'Examen des Théories et des Méthodes de Traitmens qui ont été adoptées dans ces Maladies, et principalement la Méthode thérapeutique employée à l'Hôpital Militaire d'Instruction du Val de Grace.* Par H. M. J. Desruelles, Docteur en Médécine, &c. &c.—*Paris*, 1836. 8vo. pp. 668.

   *A Practical Treatise on Venereal Diseases; containing an Examination of the Theories and Methods of Treatment which have been adopted in these Maladies, and especially the Method of Cure practised at the Military Hospital of Instruction of the Val de Grace.* By H. M. J. Desruelles, &c. &c.

2. *Practical Observations on the Venereal Disease, and on the Use of Mercury.* By Abraham Colles, m.d., one of the Surgeons of Steevens's Hospital, Dublin, &c.—*London*, 1837. 8vo. pp. 351.

3. *Procès-verbaux des Séances tenues par les Médecins de Nantes, dans la grande Salle de l'Hôtel de Ville, pour discuter la Valeur des Doctrines nouvelles, relativement à la Nature et au Traitement de la Syphilis.—Nantes*, 1835. 8vo. pp. 151.

   *Minutes of the Meetings held by the Medical Practitioners of Nantes, in the Hall of the Hôtel de Ville, to discuss the Value of the new Doctrines relative to the Nature and Treatment of Syphilis.—Nantes*, 1835.

4. *Historisch-Kritische Darstellung des Streits über die Einheit oder Mehrheit der Venerischen Contagien.* Von Dr. Friedrich Oesterlen.—*Stuttgart*, 1836. 8vo. pp. 343.

   *Historical and Critical Display of the Controversy on the Unity or Plurality of the Venereal Contagion.* By Dr. F. Oesterlen.—*Stuttgart*, 1836.

5. *Die Mercurialkrankheit in allen ihren Formen, geschichtlich, patho-*

10

*logisch, diagnostisch, und therapeutisch dargestellt von* G. L. Dieterich, m.d., &c.—*Leipzig*, 1837. 8vo. pp. 422.

*The Mercurial Disease in all its Forms, historically, pathologically, diagnostically, and therapeutically represented by* G. L. Dieterich, m.d.—*Leipzig*, 1837.

6. *Die Behandlung der Lustseuche ohne Quecksilber oder die nicht Merkuriellen Mittel und Methoden zur Heilung der Lustseuche.* Von F. W. Oppenheim, m.d., &c.—*Hamburg*, 1827. 8vo. pp. 289.

*The Treatment of Syphilis without Mercury; or, the non-Mercurial Means and Methods for the Cure of Syphilis.* By Dr. F. W. Oppenheim.—*Hamburg*, 1827.

7. *Traité pratique de la Syphilis.* Par le Baron Philip Boyer, ancien Chirurgien à l'Hôpital des Vénériens de Paris, &c.—*Paris*, 1836. 8vo. pp. 448.

*A Practical Treatise on Syphilis.* By Baron Philip Boyer, formerly Surgeon to the Venereal Hospital of Paris.—*Paris*, 1836.

8. *A Practical Treatise on Urethritis and Syphilis.* By William Henry Judd, Member of the Royal College of Surgeons in London, Surgeon in H. M. Fusilier Guards.—*London*, 1836. 8vo. pp. 610; *illustrated by coloured Plates.*

9. *A Treatise on the Venereal Disease.* By John Hunter, f.r.s. *With Notes, by* G. G. Babington, Surgeon to St. George's Hospital, &c.—*The Works of* John Hunter, *edited by* Mr. Palmer. Vol. II. *London*, 1835.

If there is one disease which more than another merits the distinction of the term *opprobrium medicorum*, that disease is Syphilis. For little less than three centuries and a half it has monopolized a share of medical literature nearly as large as all the remaining members of the nosological family put together. And what has been effected by all these labours? One of the most obvious results, at any rate, is, that an ample library might be furnished forth, consisting of volumes exclusively devoted to the elucidation of its multifarious obscurities; yet, so far is that elucidation from being accomplished, that the fecundity of the press in works relating to venereal maladies has never been so great as at the present time.

The revolutions which both theory and practice have undergone, in every point connected with syphilis, are certainly extraordinary. The treatment, especially, has vibrated between two extremes, in support of each of which success was confidently appealed to. Till within a very few years, the statements of the old non-mercurialists, if not absolutely rejected as fabulous, were, at any rate, incomprehensible to those who held the opposite doctrine; yet the very system pursued by them rose once more from its obscurity, and, for a time, reached so high a degree of popularity as to threaten the extinction of the mercurial treatment. At present, however, although some theoretical questions may be as far from solution as at any former period, in all that appertains to the grand point at issue—the best means of cure, we may with justice affirm that a gradual convergence of opinion is daily becoming more apparent. This is not, perhaps, the only example to be gathered from the history of medicine in illustration of Goethe's striking remark, that "the human mind is ever on the advance, but it moves in a spiral line."

It would be incompatible with the limits and objects of an article like the present, even to advert slightly to the various disputed questions branching out of this complicated enquiry; we shall therefore confine our attention, as much as possible, to such points as have a practical tendency. After taking a general survey of the history of syphilis during the last fifty years, which is that portion of it relating to the rise of the new doctrine, we shall arrange our materials under the two heads of the Natural History of the Disease and the Treatment, and conclude with a brief notice of the share which the several works enumerated at the head of the article may claim in the discussion, and of the information they communicate upon the different branches of the subject.

I. Into the long-agitated questions relative to the early history of syphilis, it would be foreign to our purpose to enter. Whether it arose at the latter end of the fifteenth century, as an entirely new disease, or by the graft of a disease then existing upon a group of disorders well known to the ancients, or whether it has existed in its present form from the earliest ages, is of little practical importance. Excepting by those writers who have a sort of personal interest in maintaining the last-named hypothesis, it is now generally acknowledged that no satisfactory evidence has ever been advanced in its proof; whilst the belief in the modern origin of the disease is strongly supported by historical testimony. We must protest, therefore, against the arbitrary conclusions of M. Desruelles advanced in support of the theoretical and practical views of his own party, which are grounded upon the assumed antiquity of the venereal disease, and consist in a repetition of the doctrine of Hensler, Swediaur, B. Bell, &c. His assertion, "that not only primary venereal affections, but vegetations, pustules, rhagades, dartres, and all the exanthemata now called syphilitic, were known to the ancients, and had been described before the epidemic of Naples," is not borne out by his own historical survey: whether the syphilis of the present day is identical, in all respects, with the epidemic of Naples, is another question. Passing over, therefore, the first two of the three epochs into which M. Desruelles divides the history of the disease, we shall take up the thread of his narrative at the commencement of his third epoch, and, with some assistance from other sources of information, endeavour to extract therefrom a brief sketch of its history during the last fifty years, a period in which a more correct knowledge of syphilis, and more sound principles of treatment, have been acquired than throughout the preceding three centuries.

"It was about the year 1783," says M. D., "that many practitioners, from observing facts proving that venereal maladies are capable of cure by the unaided efforts of nature, were led to a more careful study of these affections; and, consulting the annals of science, could not but be struck with the vagueness and confusion which had hitherto reigned in the treatment. Hensler, Hunter, and Jourdan may justly be considered as the chiefs of this third epoch. The first, by his learned researches into the antiquity of venereal diseases, and the second, by bringing forward his new theory, commenced the present reform. Jourdan, by making us acquainted with Hensler, contributing his own observations, and arranging the principal facts physiologically, has also promoted it."

Before discussing their opinions, M. D. recapitulates, in chronological

65

order, the attempts made, nearly in every country, to modify the received doctrine of venereal maladies. From 1517 to 1575, Poll, Massa, Fracastorius, Fallopius, Fracacianus, Fernelius, and Le Paulmier, rejecting mercury as the general method, employed guaiacum and a rational dietetic system. From 1575 to 1774, Forestus, Senertus, de Blegny, Blancard, Musitano, Boerhaave, Ritter, Balfour, Peyrilhe assert the evils produced by mercury, and either avoid it altogether or give it in a very moderate quantity. In 1779, Cockburn affirms that all ulcers will heal under a local treatment, and that syphilitic symptoms rarely follow: he proposes that mercury should be resorted to only where constitutional affections arise. In 1782, Benjamin Bell draws a distinction between urethritis and syphilis. Hensler demonstrates, in 1783, 1st, the antiquity of primary venereal diseases; 2d, denies the necessity of a specific treatment; and, 3d, quoting Boerhaave's opinion relative to mercury, "quod semper corpori nocet nec necessarium sæpe habetur," objects to it as both needless and dangerous. In 1784, Hunter produced his theory, which is now almost generally adopted by the partisans of a venereal virus and of mercury; the consideration of which we shall carry forward to the natural history of syphilis. "In the result of his labours," says M. D., "we recognize the prelude to a more attentive examination of venereal diseases." We cannot follow our author in his historical survey of the numerous writings, from those of Girtanner down to those of Mr. Bacot, in proof of the practicability of curing every variety of venereal disease without mercury. For the results of the enquiry, instituted by order of the British government, into this matter, we refer to Mr. Bacot's excellent work, p. 52-3.*

That the non-mercurial method exhibits less decided success in the hands of English practitioners, M. D. attributes to the employment of purgatives, and the use of stimulants both locally and generally, and remarks that "their pretended simple treatment is far from deserving that name, and that neither their discussions nor practice are sufficiently enlightened by physiology." This harsh censure may in some instances be deserved; but, before we yield thus much to M. D., it might not be amiss to enquire what is considered as sound physiology by him, and see the conclusions to which his principles have led him. This, however, belongs to a future division of our subject; and we shall therefore pass on to the interesting researches carried on, in the same path of enquiry, by several of the continental states, since that period.

In the year 1822, the Royal Council of Health in Sweden, having been charged by the king to conduct a series of experiments upon the different modes of treating venereal diseases, and compare their results, in order to check the abuse of mercurials in a country where the climate rendered their use so dangerous, reports from all the military and civil hospitals were ordered to be drawn up annually. These reports establish the inconveniences of the mercurial system, and the superior advantages of the simple treatment: the former method has been gradually replaced by the latter, and at present more than two-thirds of the patients are treated by the simple method. In the various hospitals of Sweden, 40,000 cases have been under treatment, one-half by the simple method,

* Observations on Syphilis. London, 1821.

the remaining half by mercury: the proportion of relapses has been, in the first instance, seven and a half, in the second thirteen and two-thirds, in one hundred. That which the Swedish physicians term *cura famis,* "cure par la diète," is nothing more than the treatment by regimen. M. D. compares it with the French and English simple method; but it is much more rigorous, and is, in fact, a revival of the practice of Fallopius and Fernelius, or rather of Palmarius.

Dr. Fricke's experiments in the Hamburg General Hospital were first made public in 1828. In four years, out of 1649 patients of both sexes, 582 were treated by a mild mercurial course, and 1067 without mercury: the mean duration of the latter method has been fifty-one days, that by mercury eighty-five. He found that relapses were more frequent, and secondary syphilis more severe, when mercury had been given; when the non-mercurial treatment was followed, they rarely occurred, and were more simple and mild when met with. Dr. Fricke has not observed caries, loss of the hair, pains in the bones, &c. where mercury has not been given; and, in all cases of caries which have come under his care, much mercury had previously been taken. In communications to M. Desruelles, from 1829 to 1833, Dr. F. states that his practice is still attended by the same results. The four following indications are the chief points insisted upon by him in his non-mercurial treatment:—The observance of strict cleanliness; perfect repose; a rigid diet; and the employment of antiphlogistics. He has treated "more than 5000 patients without mercury, and has still to seek cases in which that remedy may be advantageously employed:" we have the means of knowing that, up to the present period, the opinions of Dr. Fricke remain unaltered.

In 1833, the French Council of Health published the reports sent in by the physicians and surgeons attached to regiments and military hospitals, in various parts of France; the general results of which are, that, although primary disease will yield to the simple treatment, in relapses and secondary affections it is not sufficient; a revulsive or contro-stimulant action is required. Sudorifics, antimony, carbonate of ammonia, mercury, and salts of gold, they admit, have proved successful; but, of all these means, the different preparations of mercury have seemed to merit the greatest confidence. "It is so much the more certain, as, during its employment, patients are excluded from all other causes of excitement, by confinement, rest, and diet; the latter carried even to extreme abstinence." Some of the reports are more favorable to the simple treatment. M. Devergie has treated, between the years 1815 and 1835, at the hospitals of Gros Caillou and the Val de Grace, and in private, more than 6000 cases. The duration of the mercurial treatment has been from eighty to ninety days; of the non-mercurial, from thirty to fifty. He is not an exclusive partisan of the simple method, but adopts M. Desruelles' principles. In a report, by Dr. Rufz, of the comparative results of the two methods in 520 cases, at the Hôpital des Vénériens, in 1830, it is stated that the cure by mercury was one-third longer than that by the simple method; but it would not be just, observes M. D., from this statement to draw conclusions too favorable to the antiphlogistic treatment, as the cases treated by the former were more serious and extensive than those submitted to the latter; and this circumstance, we are convinced, has been too much overlooked in examining the results of comparative experiments. In Dr. Heisch's thesis on the treatment of venereal maladies, he gives an abstract of his observations at the Strasbourg Hospital, in 1834, under M. Kayser. Out of 486 cases of primary and secondary affections, (the latter occurring in a very small proportion,) a few obstinate affections only required mercury, and that in very minute doses. Since 1831, 5,271 patients have been treated by this combination of the two methods, and the number of relapses and secondary affections calling for the employment of mercury has been very small. No case of caries, and only one or two instances of exostosis, have been observed. Full reliance may be placed on these facts, as regiments remain in garrison at Strasbourg for five or six years.

From the records of the hospital at Rennes, under M. Rapatel, furnished by M. L. Desruelles, brother of the author, it appears that, between 1826 and 1835, 7,317 cases have been treated by the two methods, the results of which are carefully drawn up in a tabular form, and a number of conclusions appended. MM. Rapatel and L. Desruelles rely on the simple treatment in the majority of cases. In ulcers they have sometimes been obliged to resort to mercury, and have also found it useful in certain consecutive affections, prefacing its administration always by antiphlogistics and strict diet, and continuing these means throughout. The deuto-chloruret (oxymuriate) of mercury has been the preparation employed, combined with opium, in doses of a quarter-grain each. They also give the proto-ioduret and cyanuret of mercury, with opium.

In 1827 and 1828, M. Desruelles published two volumes of Statistical Memoirs, containing 1,312 observations, collected at the Val de Grace, from 1825 to 1827, on the two methods of treatment. The comparative results led him, in 1827, to confine himself to the simple treatment as a general method, and administer mercury only in certain exceptional cases. From that period till 1835, 8,810 cases have been under his care, and the results entirely accord with those obtained by Rapatel, Kayser, Fricke, &c., and the Swedish Council of Health above cited. Upon this experience he lays down ten precepts, in which the principles of his treatment, to be hereafter detailed, are distinctly embodied, and closes this portion of his work by disclaiming any intention to reject the mercurial treatment, which he believes, on the contrary, may be useful in certain cases, associated with the simple method, and in the hands of able practitioners.

To the above contributions to our experience of the effects of the simple treatment of syphilis, we may add a few experiments extracted from the work of Mr. Judd: his conclusions therefrom we shall notice in a future page.

"*Exp.* 1. Ten primitive venereal sores and buboes were taken promiscuously as they offered, and treated without mercury. After a lapse of three years, but two of the patients out of that number suffered from secondary symptoms; one of them presenting an eruption of lichen, and the other a set of maculæ.*

"*Exp.* 2. Ten other primitive cases were then taken promiscuously, and treated with small portions of mercury during nine days, and by the end of the first year they had furnished so many as five cases of secondary symptoms,—viz. an eruption of

* "Thirty other primitive venereal sores were also treated without mercury, and but five cases of secondary affections were the result of the whole of them."

puniceous patch with iritis; of lichen with effusion; of ecthyma with nocturnal pains; ecthyma with periostitis; and of herpes with cynanche.

"*Exp.* 3. Ten more primitive cases were taken promiscuously, and treated by keeping the patient's mouth sore during a fortnight: from these, at the end of two years, but a couple of secondary cases resulted,—viz. ecthyma with an exfoliation of an alveolar process, and lichen with a sore throat.

"*Exp.* 4. Ten fresh cases were next taken promiscuously, and each was treated by the mouth being kept sore during three weeks: from these, after two years, there resulted but one secondary symptom,—viz. a venereal sore throat.

"*Exp.* 5. Ten other venereal patients, taken promiscuously, had their mouths kept sore during a month each; and from all these, after a similar period, there resulted only two sets of secondary symptoms,—viz. iritis and lichen, with sore throat and nocturnal pains." (P. 537.)

We thus find, then, that the sum total of cases submitted to experiment in the above reports amounts to about 80,000; indeed, they might be fixed at a much higher number: and it appears that the proportion of relapses or secondary affections, where the primary symptoms have been treated without mercury, is, at its lowest estimate, reduced to *ten*, at its highest to *twenty*, in the hundred. We are perfectly willing to admit that the great irregularities which characterize syphilis, and the very different conditions under which it appears, give less force to statistical evidence applied to its elucidation, than to that of many other diseases; yet, making every allowance for data thus collected, and taking into consideration the incompetency of many of the individuals to observe accurately, and the party-feeling of others, together with the perplexities arising from the mal-administration of mercury, &c., it cannot be denied that a sufficient mass of observations remains to establish the fact that a large majority of primary syphilitic affections get well, like ordinary ulcers, under simple treatment, or even by the unaided powers of the constitution; and that, of those cases of secondary disease which do occur, although, perhaps, subject to more frequent relapses, the greater number will ultimately wear out or be overcome by the mere action of the secretory and excretory functions; thus leaving but a small remainder of inveterate instances to be combated by other means. Although a stanch advocate for the mercurial method, properly conducted, Dr. Colles thus expresses himself with respect to the non-mercurial system of treatment, of which, by the way, he admits that he has made but one full year's trial.

"We must acknowledge that the profession is highly indebted to those who have lately introduced the non-mercurial plan of treatment; for we have now not only acquired a second line of treatment for venereal cases, but, what is of the highest value, we have been released from an inveterate and deep-rooted error; from an unfounded conviction that the venereal disease could not be cured by the innate powers of the system, unless aided by mercury. I need not add that all the opinions and practices consequent on this prejudice have been subverted." (P. 319.)

II. If the simple or non-mercurial plan of treating syphilis had produced no other benefits, medical science must lie under eternal obligations to it for the light which it has thrown on the Natural History of this disease; and we shall now proceed to notice some of the important facts flowing from this source, as well as others, tending to the same end, derived from other quarters.

Before the time of Hunter, experiment and induction had never been brought to bear on the difficulties which beset the whole question of syphilis: it being regarded by each sect according to the philosophical theory professed by it. "His invaluable treatise," to quote the words of Dr. Colles, "poured a flood of light, not only on the natural history of this disease, but also on its pathology and treatment."

The discussion as to the immediate agent of syphilis is not a mere dispute about words, nor does it involve questions alone of a purely theoretical character, since our views of its nature and treatment are materially modified by the doctrine we may hold on this subject. Whether it be termed virus, contagious principle, or irritation, its presence can only be detected in its effects. The question therefore is, Do these effects appear to be regulated by any fixed laws, in which, making due allowance for disturbing influences, we are enabled to trace a clear chain of cause and effect, and to distinguish them certainly from other diseases? Or, do they present themselves under aspects so various and uncertain as to baffle our efforts to reduce them to any precise order, group them in one family, and derive them from any one specific source? In other words, Do venereal maladies exhibit those characters which distinguish diseases arising from morbid poisons? Or are their characters similar to those produced by common causes of irritation?

The solution of this question—or these questions—will be found in the examination of the characters of the disease, which are, it must be admitted, sufficiently various. In these varieties of manifestation, syphilis differs, as in many other points, from contagious diseases in general; and this uncertain character of its symptoms, which deprives us of much of the assistance to be derived from an appeal to analogy, has been one of the most fruitful sources of dispute. While one party contends for a single contagious principle, ascribing the diversity of forms in which it manifests itself to idiosyncrasy, climate, habitude, age, greater or less intensity of the virus, mode of its absorption, &c., certain writers have attempted to trace this diversity to a corresponding variety of poisons; as to the precise number of which, however, no two of them are agreed. Under these circumstances, it is not surprising that a third party should have sprung up, denying that we have any just grounds for deriving from a specific virus of modern origin, or including under one head, an assemblage of disorders of various kinds, to which the human race has always been subject.

The first attempt to distinguish diseases of the sexual organs arising from impure connexion, from those produced by other causes, was made by Mr. Hunter, whose views were most zealously adopted by Mr. Abernethy. The division, by these two observant writers, of this class of diseases into syphilitic and syphiloid, or pseudo-syphilitic, originated principally in an impression that syphilis "had no tendency to cure itself, or that the constitution was unequal to the cure of the disease;" which is but another mode of expressing their conviction that mercury was the sole and indispensable remedy. The fallacy of this ground of distinction being now fully recognized, the so-called syphiloid and pseudo-syphilitic affections resolve themselves—(1) into cases of syphilis, which, proving intractable under the mal-administration of mercury, were allowed to get well without it; (2) forms of that disease which did not happen to correspond with arbitrary definitions; and (3) other diseases which have been improperly confounded with syphilis. The knowledge, acquired chiefly by the medical officers serving in the Peninsula, that a large proportion of primary venereal affections were curable without mercury, modified the doctrine of Hunter and Abernethy, and led to the researches of Carmichael, Evans, and others, who were still impressed with the idea that genuine syphilis absolutely required the specific remedy. The results obtained by these gentlemen, too hastily generalized, tempted them also to lay down arbitrary distinctions which subsequent experience has not confirmed; or, rather, it has suggested the probability that several of their varieties were the same lesion observed at different stages and under different aspects.

These distinctions gave birth to the theory of a plurality of venereal poisons; an opinion which is carried to an extreme length by one of the authors under review. Mr. Judd believes that the venereal virus originated in a disorder (the pseudo-syphilis of Mr. Abernethy,) generated by promiscuous intercourse, the acrid matter of which, when brought into contact with an abraded surface, produced genuine syphilis. As this genuine syphilitic virus consists, at the present day, of the union of an indefinite variety of morbid poisons, there is no difficulty, according to Mr. Judd, in accounting for the variety of primary and secondary affections resulting from the contamination of a single primary sore.

"To illustrate this by a tangible and more familiar example," says Mr. Judd, "first, let us suppose a person with a pimple on the glans has connexion with a prostitute; secondly, that another with a vesicle has commerce with her, and likewise a third with a pustule or an open sore. Now, we will suppose there lies a collection of poisonous fluids in her vagina, each capable of producing its own peculiar form of disease so soon as it is placed in the living body; and this is that sort of mixture that we are in the habit, from prejudice and from its being generally inoculated through one sore, of considering as consisting of but one poison; whereas, in reality, it is a mixture of poisons, or a compound amalgam of modified ones. Next, let us take vaccine lymph, and mix it with varioloid pus, and both with herpetic fluid, and then I think you may imagine you are approaching to (though wanting many ingredients of) the compound venereal virus of the present day." (P. 149.)

Mr. Judd enumerates nine varieties of primitive affection, from "a superficial redness" to the "phagædenic bleeding ulcer," all of which, he says, are followed by secondary disease; but he specifies no diagnostic marks by which they may be distinguished from similar local maladies arising from ordinary causes.

It remains to be seen whether the disciples of the new doctrine have been more successful in solving this difficulty, since they claim an emancipation from the prejudices flowing from the idea of a specific disease and a specific remedy. In this enquiry we shall take M. Desruelles as the representative of the class to which he belongs.

After objecting to the existing definitions and classifications, M. D. proceeds to divide venereal maladies into primary, secondary, and consecutive. The first and third correspond with the primary and secondary diseases of former classifications; the second class comprises bubo and similar affections intermediate between the two others. The second and third classes may depend on the absence of all treatment, on incomplete treatment, or on a simply local treatment of the primary affection.

The forms and characters of these diseases vary according to the organic conditions of the tissues affected, the manifestations of vital action, and the mode of application of the contagious cause. They may be arranged under the erythematous, the ulcerative, the phlegmonous, and the vegetative forms.

The erythematous form, accompanied by purulent secretion, is (according to M. D.,) the origin of all the others, and the most frequently met with. Where the irritation is equally spread over the surface of a tissue, without concentration or confinement, it is never followed by ulcer or abscess, but by injection of the minute vessels, with an increase of the normal secretion, and generally terminates in resolution. Urethritis, balanitis, posthitis, and vaginitis, (inflammations of the urethra, gland, prepuce, and vagina,) are examples of this. When the irritation is concentrated on one or more points, insinuates itself into the sebaceous follicles, or in any way penetrates beneath the surface, small purulent cysts are developed, the pustule breaks, and ulceration is produced. But ulceration is not exclusively dependent on venereal causes, nor is it peculiar to the genital organs; and what character has the venereal ulcer to distinguish it from that produced by other causes? M. D. asserts that it has none, citing in proof of this the experiments of Dr. Fricke and others, in which ulcers, bearing all the characters of the Hunterian forms, were developed by placing, for twenty-four hours, between the prepuce and the gland, a grain of deuto-chloruret of mercury, a piece of agaric or any other irritating substance. Mercury, he continues, was once looked upon as a touchstone of their venereal origin, when that was uncertain; but a recourse to such a diagnostic in the present day would be absurd. If the history of the supposed origin of these diseases did not settle the point, inoculation has been resorted to: against this test, however, M. D. joins Cullerier and Ratier* in protesting strongly, at the same time that he avails himself of M. Ricord's experiments on the subject, which coincide, in the main, with those of Mr. Wallace, to be noticed hereafter. From all these premises, M. Desruelles concludes "that there is but one primitive kind of venereal disease, the *erythematous;* that no venereal disease possesses characters sufficiently distinct to indicate infallibly the nature of its cause; and that inoculation does not throw sufficient light on this matter to dissipate all doubt."

If the characters of syphilitic primary affections are not sufficient to enable us to distinguish them with certainty from similar affections arising from ordinary causes, neither do they afford us sure grounds for any practical distinctions between each other. The only circumstance in which all writers, from Astruc and Hunter downwards, agree is, that ulcers of an obstinate disposition, attended or followed by induration, are those most liable to be succeeded by constitutional disease; but the conclusion that the indurated form alone characterizes genuine syphilis, according to the dicta of Hunter, Evans, and Carmichael, were it true, would confine syphilis within very narrow bounds; for the genuine Hunterian chancre is now extremely rare. Hunter, in point of fact, abandoned his own definition in practice; Astruc laid quite as much stress on the epithet *contumacia* as on *callosa*, applied to chancres; and we think all candid practitioners will allow that the following opinions and definitions of Dr. Colles are much more consonant with general experience than the partially accurate but too confined views of John Hunter.

* Dictionnaire de Médecine et de Chirurgie pratiques. Art. *Syphilis.*

"Although every surgeon must admit that Mr. Hunter's description of a chancre is correct, and drawn from nature, still I believe few will confine this term, or that of primary venereal sore, to those ulcers only which answer to his description. As the result of long, attentive, and anxious observation, I should say that primary venereal ulcers present an almost endless variety of character. I would define a primary venereal ulcer to be 'one which is remarkably slow in yielding to ordinary, mild, local treatment, but which is curable by mercury, and which, if not so cured, is likely to be followed, in two or three months, by secondary symptoms, which again also are curable by mercury.' If, then, there be, as I affirm there is, an almost endless variety in chancres, how can we decide on the nature of primary ulcers, so as to pronounce some to be syphilitic, and others to be mere common sores or simple excoriations? I reply, that we are to be guided in our decision by observing, first, that many of these suspicious ulcerations cannot be referred to any class of common ulcers, as they strikingly differ from them; and, secondly, by attending to the course which these take, when not interfered with by any stimulant or caustic application, and when treated only with some mild ointment or cold water. If, under these circumstances, we find that, after eight or ten days, such ulcers show no disposition to heal, and if at the same time there be a total absence of any cause, such as defect in the general health, to account for this obstinate condition of the local disease, we may then pronounce them to be syphilitic." (P. 75.)

The secondary or constitutional effects of the syphilitic infection are, if possible, more variable and uncertain in their characters than the primary. And here we would remark, that we are more disposed to agree with those writers who place bubo among the primary symptoms, than to assign it a separate class with M. Desruelles. It is, in fact, an accidental affection, depending merely on anatomical structure and function of the parts liable to it, and neither deserves the prominent station to which he has raised it, nor the importance bestowed upon it by M. Boyer,—that of being the diagnostic mark of genuine syphilis.

As in the primary, so also in the secondary symptoms of the venereal disease, a correct knowledge of their natural course and termination was long retarded by the belief in their intrinsically destructive character, and the necessity of a specific remedy to overcome it.

"It was Hunter," as Dr. Colles observes, "who first demonstrated with clearness and precision that secondary symptoms succeed the primary at an interval of six or seven weeks, and that they are generally preceded by an eruptive fever, which is ordinarily of an inflammatory type: he also described the third order of symptoms; and all this he has done with an accuracy and fidelity which the subsequent experience of the profession has amply confirmed." (P. 3.)

Dr. Colles laments that Mr. Hunter "did not prosecute his enquiries still further; that he did not, for example, trace the full progress of secondary symptoms, or mark their natural tendency to amend, and then again to relapse, each unfavorable change being preceded by constitutional disturbance; and that he has omitted to notice the probable period of time which these symptoms occupy, from their origin to their acme, and thence to their decline." There can be no doubt that Mr. Hunter's further enquiry was arrested by the impression above alluded to; consequently, that he was not justified in withholding mercury after the distinct development of secondary symptoms. "But," says Dr. Colles, "does it not appear strange that subsequent writers have not made some effort to supply these deficiencies?"

It is true that no writer, as far as we can recollect, has done so in the systematic manner which Dr. C. requires; but we shall find that much information has been stored up on this stage of the course of the disease, and it will be our business to enquire how far it corroborates the following observations of Dr. Colles.

"I have observed, that when the local symptoms have become fully established, which is probably in two or three weeks after they make their appearance, they then become stationary, and the constitution is relieved from the febrile disturbance. In this quiescent state the symptoms may remain for about three weeks, they will then show a strong disposition to amend, and sometimes they will proceed so far, as to impress the surgeon with a hope, and the patient with a firm conviction, that he is about to get perfectly well. But this illusion, having lasted for two or three weeks, is dispelled in general by a fresh attack of eruptive fever, and by an eruption or sore throat, although sometimes other symptoms, (iritis for example,) be added to those under which the patient had been previously suffering. How long the disease might continue in this condition of alternate improvement and deterioration, I cannot pretend to say, as I have not had an opportunity of witnessing in the same individual more than two, or at the utmost three, of those revolutions: from what I have seen of such cases, I am led to say we may calculate upon each relapse as likely to recur every third month. These remarks apply to the secondary symptoms, as observed for two, three, or four of the first revolutions. At a later, and sometimes, though rarely, in an early stage of the disease, we find a somewhat different process ushering in a new attack; thus we sometimes observe that the patient who during the four or five preceding weeks had been improving in flesh, colour, appetite, and strength, now begins to exhibit a different aspect; his countenance alters considerably, it becomes sickly, his complexion assumes a waxen hue, and he evidently loses flesh from day to day, and all this takes place whilst the patient himself is not at all aware of the change, and is still less suspicious as to its cause. In some time, however, the patient complains of night sweats, want of sleep, loss of strength, and declining appetite, so that in the course of two or three weeks he is reduced to a state of great weakness and emaciation: this downward course proceeds steadily until a fresh order of symptoms appears and becomes established." . . . . .

"After an attack of this lengthened nature we seldom if ever observe a distinct eruptive or premonitory fever appear in this patient; and I may also add that the local syphilitic symptoms cease to present their characteristic signs as strongly marked as heretofore. Thus it would seem as if each eruptive fever, or rather each succeeding attack, brings the constitution into a weaker and weaker state. In the very advanced stages of the venereal disease we do not see those periodical changes; the constitution then appears unequal to any struggle, so that one continued and increasing state of debility, with slow fever and great emaciation, are conjoined to the local symptoms, while the latter also are but little disposed to undergo any change, except a slow and gradual deterioration: thus, I have sometimes, though rarely, seen a tubercular eruption, combined with pains of bones and joints, continuing during four or five years, and undergoing no material change, being at one time a little better, and at another a little worse." . . . . . .

"When the disease has arrived at that advanced stage in which the general health has been broken down, and the local symptoms are so much changed as scarcely to be recognized as venereal, it is still a matter of uncertainty how long the patient (if not relieved by art) is to drag on a miserable existence, or in what manner a termination is to be put to all his sufferings. Many of its unfortunate victims are destroyed by what may be considered a continuation of the disease; but by far the greater number appear to be carried off by other complaints to which we may presume they were naturally disposed, or to which they were rendered liable by the very weakened and reduced state of their general health." . . .

"From this account then it is very evident that there is no certain period after the appearance of the primary disease at which death determinates such protracted cases of syphilis. We may say that in general this event takes place between the second and fifth or sixth year." (Pp. 8, 10, 11, 12.)

In proof of the naturally protracted duration of the disease, Dr. Colles selects two strongly-marked cases from many of a similar character, (see p. 194,) and in his Chapter on the administration of Mercury, where he ascribes the masking of the syphilitic symptoms to a mismanagement of mercury, or to its timid employment, he adds that although a very few may get well under the non-mercurial plan "many more have been allowed to sink into an untimely grave, by the slow and silent, but certain operation of the venereal disease, the symptoms of which have become so changed or masked that common observers could not recognize the features of the original case." Dieterich refers the masked syphilis of the writers of the sixteenth and seventeenth centuries to the abuse of mercury, and not to a combination of the effects of mercury with the syphilitic poison, an idea entertained by Hunter, but in which, as Dieterich shows, Paracelsus had preceded him. His arguments to prove this, however, are far from satisfactory, since there is, unquestionably, a marked distinction, between the diseases arising from an undue administration of mercury for the cure of syphilis, and those common among workmen exposed to the fumes of mercury, as well as the effects produced by its too copious use in other diseases. "Since 1816, (says Oesterlen,) Biett has annually passed in review from five to six hundred individuals impregnated thoroughly with mercury in the prosecution of their business (gilders, pewterers, &c.) and has never remarked in them the slightest affection of the bones, &c. They come in crowds to the Hôpital St. Louis in order to use the steam-baths for the relief of mercurial palsy."

That this masked syphilis, as a natural character of the disease, is so common as Dr. Colles believes, we are very much inclined to doubt. Where the health is undermined, and death is produced by morbid changes in the vital organs, supervening on protracted syphilis, we think the explanation of Mr. Hunter more rational, viz. that "the venereal disease often becomes the immediate cause of other diseases by calling forth latent tendencies into action."

Every person, as Dr. Colles observes, who has paid even moderate attention to the secondary forms of the venereal disease, will admit the accuracy with which Mr. Hunter has described the order of parts successively attacked. Exceptions to his rule occasionally occur, which derangement, Dr. C. ascribes, in some cases, to the mal-administration, in others to the excessive use of mercury. The form of venereal ulceration of the throat, described by Hunter, "may be looked upon as the type of the genuine venereal sore throat; still we must not rely (solely,) on the present appearances, however strongly marked, but should trace back the previous history, and enquire into the treatment of the primary disease;" and this advice is applicable to the management of all varieties of secondary syphilis.

A vast variety of ulcerated throats, in a descending series, will be seen between this Hunterian sore throat and the aphthous-looking ulcer, frequently the appearance put on by the relapsed sore throat. These varieties depend, doubtless, on idiosyncrasy, treatment, &c. and the surgeon must exercise his judgment in their management. Unless we take the history and previous treatment as our guide, we shall find it difficult to distinguish between many of these affections of the throat, ulcers and fissures of the palate and tongue, ozæna and iritis, arising from syphilitic infection, and those from scrofula and other causes.

With respect to venereal eruptions, Dr. Colles does not believe that they are at all characteristic of distinct forms of syphilis; 1st, he has not been able to trace back particular forms of eruption to particular forms of primary ulcer; 2d, he has not unfrequently observed varieties of eruption existing together in the same individual, the papular and pustular, for instance; 3d, after the removal of the first eruption by mercury, or by other means, the second crop will frequently prove of a different kind; thus the papular will be succeeded by the pustular variety; and, finally, any form of eruption may be converted, by injudicious treatment, the excessive use of mercury in bad habits for example, into one which is most obstinate and severe, that of rupia, namely. In proof of this irregularity, there is no lack of evidence. Speaking of Mr. Carmichael's classification, Oesterlen observes, that "experience shows that no constant connexion exists between his primary and secondary affections." He refers to Hennen's work, particularly to a case in which nearly all Mr. Carmichael's varieties of eruption followed a Hunterian chancre, and quotes his conclusion, which is similar to the opinion expressed by Mr. Samuel Cooper, that "from one and the same ulcer may follow eruptions of an entirely different character, whilst from totally different ulcers may proceed one and the same affection of the skin." Mr. Bacot also shows the contradictions and inconsistencies of this classification, and adds that contemporary practical surgeons have sought in vain to follow it at the bed-side. " Biett, notwithstanding all his acuteness, (says Oesterlen,) has found the classification and marked diagnosis of syphilitic cutaneous affections a most difficult matter; else why should the appearances at different stages form the ground of his classification of all other diseases of the skin, whilst the source alone forms that of venereal eruptions, which are thrown together under the term syphilides? Cazenave and Schedel also in vain strive to demonstrate the possibility of a sure diagnosis."

These objections to classification, with our present ignorance of the laws to which syphilitic consecutive affections are subject, apply, at least with equal force, to that of Mr. Judd. In the face of them, however, he insists, at p. 171, " on nature's universal plan of formation and reproduction by the like of its kind," and is of opinion "that the primitive pimple, vesicle, pustule, or sore, (if it contaminates,) invariably leads to and produces an eruption of a similar kind, and that this sort of virus, after connexion, again produces eruptions in others of a like sort, and so on ad infinitum." At the same time, he admits that increased degrees of irritation will convert a primitive pimple into a vesicle, and the latter into a pustule,* which modifying influence aids him further in explaining apparent discrepances with his theory. Having estimated the primitive syphilitic affections at nine, he divides cutaneous eruptions into the same number; the following passage reveals the process by which this problem was worked out.

"In limiting the number of sores and primary affections to about nine, (but without including their modifications,) I do imagine that I have come very near to the true number met with in England, Scotland, and Ireland: for, from the numerous drawings I had made of sores, and sketches of various venereal eruptions, and from the

* Biett's experience is opposed to the opinion that primary sores originate ordinarily by a vesicle or pustule ; Cazenave states that they commence " the most commonly by a red spot, a true ulcerative inflammation, without elevation of the epidermis."—Rev.

many manuscript accounts I have collected during a period of near twenty years, I can assert with truth, that on summing up all the various appearances of the sores, and comparing them and the number of the eruptions, I actually found their numbers exactly corresponded. At this I rejoiced, and hailed it as a happy criterion, showing that I had approached as near to the true number as the difficulty of the subject, the features of sores, and the variety of cutaneous affections probably admit of." (P. 185.)

However gratifying this coincidence may have been to Mr. Judd, the whole classification, it is plain, is perfectly arbitrary; if we are to derive each variety of secondary from a corresponding primary affection, and both, with the author, from a distinct poison, there is no reason why they should be limited to nine, or even twenty, since the varieties of both forms are sufficiently numerous to warrant it; at any rate, the practical results would still be much the same.

Mr. Babington declares the variety of venereal eruptions to be so great as to baffle description. The more distinct forms, he thinks, may be arranged under the four heads of Tubercles, Lichens, Psoriasis and Lepra, and Rupia. The most important practical point, as nearly all writers agree, arising from these distinctions, is, that rupia and ecthyma are met with in conjunction with a highly dangerous general depression of the health, and require the greatest caution in their management.

On the subject of nodes, pains in the limbs, hectic fever, and the later phenomena of syphilis, we shall speak when treating of the use and abuse of mercury, from which cause they more frequently result than from the unmixed action of the venereal infection.

On one class of venereal disorders, congenital syphilitis, namely, which differs in some particulars from the diseases with which we have hitherto been occupied, Dr. Colles furnishes much interesting information. "The phenomena, he observes, of the syphilis infantum, or hereditary form of the disease, deviate in so striking a manner from those of the disease as it affects adults, that some authors have not hesitated to deny its venereal origin." Most writers are now agreed in the opinion that it is communicated by the mother during utero-gestation, and not during parturition; consequently, that it is an instance of the transmission of secondary syphilis. The disease may exist at birth, or may not appear for some days, or weeks, after birth. It is indicated by copper-coloured spots about the anus and genitals, running into excoriation; a peculiar shrill, hoarse voice; ulcerations and excoriations of an aphthous character at the angles of the mouth, and on the tongue, palate, and throat; and, in its more advanced stages, obstruction of the nose, emaciation and a senile expression of countenance, enlargement of the glands and general cachexia, leading on to death. The child may also be infected after birth, by a nurse suffering under syphilitic ulceration of the nipple, or by its mother under the same circumstances, if the disease of her nipple has been derived from a strange child; but no instance is known of a child infecting its own mother, although it will immediately communicate disease to a strange nurse: of all these circumstances Dr. Colles relates instances.

The infection of the nurse manifests itself in an ulceration of the throat, which cannot be distinguished from that succeeding primary disease, cutaneous eruptions, and the formation of moist excrescences about the pudenda, which are capable of infecting her husband, in whom they are, likewise, followed by constitutional effects. In him the ulceration of the throat has not the same true venereal character; it is superficial, and its surface is covered with patches of whitish lymph. Dr. Colles believes that the disease may be further imparted to other members of the family by contact, use of the same utensils, &c. for it is remarkable, he adds, that its contagious property increases in proportion as it extends further from its source. Its symptoms, in different individuals, bear an exact resemblance to each other, which is not the case in the ordinary syphilis of adults. In this third remove, too, from the source of contagion, it is permanently fixed in the parts it first seizes and is of a much milder nature. Hunter believed that secondary affections could no longer infect; but, as Mr. Babington justly observes, "it is impossible to admit Hunter's argument that secondary syphilis never contaminates. The facts are so well established, that it is more easy to question the principle than to doubt the facts." No one, who has seen much of venereal diseases, will now dispute this. An interesting case of disease thus produced is given by Dr. Colles (p. 265), the symptoms of which tally remarkably with those of the disorder communicated to the nurse by a diseased infant. Whether the contagious principle of the disease passes through several grades of decreasing intensity, as Dr. Colles is disposed to believe, or whether the peculiar deviations, described by him, depend upon complications, or modifying causes, of which we are ignorant, we shall not attempt to decide.

On the whole, then, we think a sufficient analogy with specific diseases has been made out to justify us in classing venereal maladies among them. "This circumstance alone," observes Oesterlen, "bears us out in deriving syphilis from a specific source; that a certain form of disease, if it does not reproduce precisely the same form in the next individual, will do so in the third or fourth." Although this author will not venture to decide the question of the unity or plurality of the morbific agent in syphilis out of the vast mass of conflicting testimony he has sifted, the above argument, as well as the preponderance of authority throughout his work, is distinctly favorable to the belief in a unity of poison. That this one virus undergoes modifications in its course is, also, rendered very probable by many of the facts we have detailed. If the results of Dr. Wallace's experiments on it, by inoculation, are verified, and there is no reason to doubt their accuracy, we may set this down as positively determined.*

Assuming, then, a morbid poison as the immediate cause of syphilis, in opposition to the doctrine of the Broussaisists, to what does our actual knowledge of the remaining chain of effects, which constitute the natural history of the disease, amount? Examining the links of connexion, we shall find them placed, with occasional exceptions, in the following order: At an uncertain period, within given limits, after intercourse with a person capable of communicating infection, of which power the results are often the only absolute proof, an erythematous excoriation, or an ulcer, may appear on the sexual organs. This excoriation or ulcer, termed the primary affection, does not, *per se*, exhibit characters which will *infallibly* distinguish it from similar affections arising from ordinary causes. Its syphilitic character is to be inferred, with more or less probability, from

* See his excellent Lectures in the Lancet.

its aspect and an intractable disposition, which probability is much increased by the foregoing suspicious connexion, but can only be positively established by the subsequent occurrence of certain other affections, termed constitutional, which develop themselves comparatively seldom, but often enough to authorize us to conclude that a relationship of cause and effect exists between them. These constitutional symptoms have the property of affecting certain parts in a regular order of succession, not, however, constantly followed, or of breaking out in the same parts, with more or less frequency according to the peculiar constitution, habitude, management, &c. If these modifying circumstances are unfavorable, the symptoms proceed, in a series of recurring exacerbations, from bad to worse, till they are arrested by the only remedy which has control over them, or till death from cachectic fever and exhaustion, attended, sometimes, by other morbid complications, closes the scene: but, in the majority of instances, these unfavorable modifications not being present, the constitutional affections gradually wear themselves out, a consummation which may be considerably forwarded by rigid attention to Hygienic rules, and a judicious administration of mercury.

III. It is fortunate that upon the most important branch of the subject, *the Treatment*, the opinions of practical men do not vary so much as on theoretical points; and there is good reason for the belief that the debateable ground is daily becoming narrower. Allowing for shades of difference, two distinct systems of treatment, only, can now be specified. According to the first, both primary and secondary symptoms are invariably treated, under ordinary circumstances, by simple means, and mercury is kept in reserve for obstinate and intractable cases alone. The second method takes as its principle, that the occurrence of constitutional affections ought never to be permitted; and to prevent this, mercury is given at the onset, and continued until the surgeon feels perfectly secure that there is no danger of such a result. Into some details respecting each of these systems, we now propose to enter, in order to ascertain, if it be possible, which of them has the best claim to the merit of removing syphilis *tuto*, *cito*, *et jucunde*.

1. The simple method of treating syphilis, is divided into the internal or medical, and the external or surgical. The former consists of two parts; the observation of certain hygienic rules, and the employment of general therapeutic means. The diet must be light and mild,—meat, and all stimulating viands retarding the cure; even with the lightest diet, the hunger must never be quite appeased. As the cure advances, the quantity may be increased, but not until it is complete is even a gradual return to the ordinary regimen to be allowed. In proportion to the intensity and duration of the disease, to the youth, vigour, and excitable character of the patient, must be the austerity of the diet. Diluent beverages, decoctions of barley, liquorice, and linseed, alone or mixed with milk, should be taken freely; to the amount, indeed, of several pints each day. In summer, lemonade, orangeade, &c. may be substituted. Perfect repose must be ensured by confinement to the bed. Constipation must be obviated by the use of emollient clysters or mild laxatives. Purgatives, however, require caution in their administration. The air should be pure, and maintained at the same temperature; this is an indispensable precaution in chronic, consecutive, and mercurial affections. Exercise is only to be recommended in the convalescent stage; it is then a valuable auxiliary, combined with country air. In chronic syphilis, it may be often carried to fatigue with advantage. As the passions frequently lead to an aggravation of venereal diseases, by interfering with the necessary calm and repose, tranquillity of mind must be strenuously insisted on. Tepid baths, repeated every four or five days, are always attended with benefit. General bloodletting is often required where the primary disease is intense, or the system excited, and the patient plethoric; but is injurious when indiscriminately adopted.

In instituting the simple external or surgical treatment, strict attention to cleanliness, and the position of the diseased parts, is never to be lost sight of. Emollient decoctions as fomentations, or dressings of simple cerate, are the best applications; and the dressings should not be too frequently renewed. Leeches are generally necessary, and are more serviceable when applied on the diseased part than in its neighbourhood. A succession of leeches, few in number, but frequently repeated, so as to keep up an almost constant flow of blood, (*saignée permanente*), produces the most beneficial results. In cases of ulcer their application is most efficacious to the ulcerated surface; and the inconvenience occasionally produced by the leech-bites taking on the same action, is thus avoided. The same rule applies to their use, in consecutive ulcers of the nasal fossæ, and soft palate. The greatest benefit is derived from the external use of a concentrated solution of opium (in the proportion of about ʒij. to ℥j. of water); it soothes excessive irritability in all cases. M. Desruelles has frequently seen vegetations entirely removed by it. When the suppuration is moderated, and the surface of the ulcer cleansed, stimulating dressings, consisting of solutions of the sulphates of alum and copper, the nitrate of silver and subacetate of lead, favour cicatrization.

Although the simple treatment, traced out above, suffices generally for the cure of primary and consecutive venereal affections, it is not the sole treatment by which they may be combated. But, whether the revulsive method be employed in the first instance, or resorted to at a later period, it is established, says M. Desruelles, by the fullest experience, that the simple treatment should form the basis of a rational practice; otherwise, the patients are exposed to more serious dangers than the venereal affections themselves.

The mercurial treatment of syphilis is regarded as revulsive. This may be either external or internal. M. Desruelles is opposed to frictions, whether by the mercurial ointment or that composed of the proto-chloruret and deuto-chloruret of mercury. Baths, containing the deuto-chloruret, are dangerous. He objects to fumigations by cinnabar generally and locally, that they irritate the skin, and that the amount of absorption cannot be regulated. Frictions and fumigations, under proper precautions, are still occasionally advisable.

Among the mercurial preparations exhibited internally, the black oxyde, or soluble mercury of Hahnemann, has numerous partisans in Germany, and often produces good effects. The proto-chloruret is given, at the Val de Grace, combined with extract of cicuta, in pills containing, of each, one grain. At the commencement one or two pills are given every morning, and their number is gradually increased, up to twenty, twenty-four, or thirty daily. The medicine acts by inducing diarrhœa and salivation; and must be abandoned if the former becomes profuse. It is most useful in chronic inflammation and ulceration of the throat, and in chronic enlargement of the testicles. United with opium, and superadded to the antiphlogistic treatment, it cannot be too much recommended. Biett employs the same treatment in secondary syphilis, taking care, however, to avoid salivation.

M. Desruelles administers the deuto-chloruret in the forms of tincture and pills. Of the first he gives a portion containing one-eighth of a grain, combined with three-eighths of a grain of opium, in decoction of marshmallow, every morning, and gradually increases the dose to half a grain of the oxymuriate, and three-quarters of a grain of opium; continuing it according to its effects. The pills contain a quarter of a grain of the former, and half a grain of the latter, and the dose is increased to double that strength. This preparation is particularly efficacious against ulcers with a hard base, which have resisted the simple treatment, or have been but imperfectly modified by it. Ioduret of mercury was proposed, in 1820, by M. Coindet, in venereal affections complicated with scrofula, and has been since taken up by Biett, as a remedy in chronic and constitutional syphilis. The proto-ioduret and deuto-ioduret are more stimulating than the chlorurets, and demand the greatest caution in administering them. The cyanuret of mercury has been recently again brought forward by Parent, who believes that venereal diseases disappear more rapidly under its influence than under that of any other mercurial preparation. He employs it in the forms of tincture, solution, pills, gargle, and externally in an ointment. Its effects are more prompt and energetic than the oxymuriate, and it is not so easily decomposed.

In his practical reflections on the action and use of mercury, appended to this portion of his work, M. Desruelles observes, that, although he is now convinced, that tumefaction, and induration of the periosteum and bones, may be produced by the venereal irritation alone, yet, those who abstain from mercury altogether, or to whom it is exhibited with great reserve, will rarely be attacked by such symptoms. "No conscientious observer at the present day, however, would venture to assert, that the simple treatment will prove a perfect security against affections of the periosteum and bones." He admits that certain ulcers, as those known by the name of the Hunterian chancre, the phagedenic, and such as leave an indurated cicatrix, are more frequently followed by consecutive symptoms, when treated simply, than when mercury has been properly used. He therefore adopts the following line of practice: in the early stage, if there is no reason to believe that the venereal influence has spread beyond the genital region, he insists on the strict observance of the simple treatment, and prohibits mercury. On the other hand, after having quieted the general system, if the ulcers have existed for twenty or thirty days, if their period of incubation has been short, and they maintain an unhealthy aspect in despite of the simple treatment, he has recourse to mercury, preferring the forms mentioned above.

Consecutive ulcerations of the soft palate and nasal fossæ he treats by antiphlogistics, merely as a preparation for the "revulsive" treatment under which he has found them most tractable, namely, the pills of calomel and cicuta, with antimonials; and, as local means, astringent and opiate gargles, and the solution of nitrate of silver. When these diseases coincide with pains and affections of the bones, the macertating practice, "cure par la faim," is indicated, and mercurials contra-indicated: opium and extract of henbane are preferable to it; yet M. D. has known exceptions to this rule, in which such affections have vanished as if by enchantment under the influence of mercury.

The objection raised against the efficacy of the simple treatment, on the score of its being attended by a greater number of relapses, M. Desruelles affirms to be unjust. As they occur after every method of treatment, the comparative frequency can only be determined by an appeal to facts, which, he contends, are in favour of the simple treatment. He concludes the enquiry by the following recapitulation of the opinions of M. Cullerier regarding the new doctrine, extracted from M. Lucas Championnière's practical researches on the treatment of syphilis.

"1st. That the relapses, after the employment of the simple treatment regularly administered, are extremely rare; but that they occur at a very early period after the primitive infection; 2d, that those after primitive symptoms, abandoned to themselves, or of which the cure has been accelerated by cauterization, are not rare, but that in general, they are not very serious; 3d, that the relapses after incomplete mercurial treatment, are very common; and that consecutive symptoms of all kinds and of all degrees of severity manifest themselves at every period; lastly, that the relapses among individuals who at every appearance of primitive symptoms have undergone a mercurial treatment in a manner the most complete, amount to a fourth part in the sum total of those he has observed; that they are excessively severe, and, almost always consist of affections of the fibrous and osseous systems, chronic tubercular affections of the skin or extensive ulcerations of the mucous cavities."

To the examination of the claims of other reputed anti-syphilitics, M. Desruelles devotes a section. He believes that gold does, unquestionably, possess a high modifying power; and that in all cases where the nature of the syphilitic affection, and its long action on the constitution call for such an influence, prompt and energetic in its effects, gold may be advantageously resorted to.

Guaiacum is little employed at the present day, except in combination with sarsaparilla, which is preferred to it. The latter forms the basis of all the pharmaceutical preparations which are now recommended against chronic venereal maladies and mercurial affections.

Those who seek for further information on any or all of the remedies which have been proposed as anti-syphilitics will find it in the work of Dr. Oppenheim, of Hamburg, the sixth on our list. In his appendix Dr. Oppenheim refers to the advantages derived from the internal use of iodine in many cases of ulceration of the throat; and as this medicine, in one of its forms, has lately acquired some celebrity as an anti-syphilitic, we shall here say a few words on the subject. The author of one of the works at the head of this article, Mr. Judd, announces, in his title-page, a substitute for mercury, which proves to be the hydriodate of potash, administered internally in doses of five grains, continued, for some time, two or three times a day. Three cases, two of them chronic syphilitic affections attended by nocturnal pains, treated in 1832-3,—the third a cutaneous eruption, constitute the amount of his recorded experience in the successful employment of this remedy; and yet he observes regarding it, "a few more such cases in addition to those

5

already detailed, are all in which the author has as yet employed this new remedy for venereal disease: but if its curative properties in syphilis are such as they appeared to be in the inveterate disease of D—— N——l, perhaps no future loss of nose or palate will be permitted to occur in England:" a sanguine conclusion this, from such slight premises. Mr. Judd, might, we think, have endeavoured to strengthen the claims of his remedy, by some allusion to the experiments of Dr. Wallace on its effects, which have been from time to time detailed in the Lancet. In a Lecture, delivered January 19, 1836, (see *Lancet*, February 6,) that gentleman tells his pupils, "This makes the 124th case of secondary syphilis which I have so treated and carefully noted. Two years and a half have now passed since I commenced the investigation, and I have collected as great a body of facts as have ever, perhaps, been collected respecting the treatment of any one chronic disease by a particular remedy." In much earlier lectures he brings forward his opinion of its value and mode of action; therefore Mr. Judd has as little excuse for his silence with regard to Dr. Wallace's labours, as claim to originality in his proposal.

That the beneficial effects of iodine in a cachectic state of system are very great, and that, in the form of hydriodate of potash, it can be more conveniently exhibited than in that of tincture, is not to be questioned; but that, therefore, it is to be hailed as a specific in syphilis is very far, we think, from being established.

2. Before entering upon the consideration of *the mercurial treatment*, properly so called, it may be well to enquire what the advocates of this system advance generally in its defence.

In the first place, then, although fully admitting the poisonous effects of mercury improperly administered, modern mercurialists deny that it is either just or honest, on the part of the advocates of the new doctrine, to accuse the remedy itself of the evil consequences which have arisen from its indiscriminate and profuse employment,—from its abuse, in short. Commenting on this injustice, Dr. Oesterlen exclaims pathetically, "'Ingratitude is the world's wages,' mercury, too, may sigh: 'because they have misused me, I now have to bear the blame!'" "Nothing," said Mr. Hunter, with something of the same feeling, "can show more the ungrateful and unsettled mind of man than his treatment of this medicine."

Secondly, in answer to the charge that the employment of mercury for the cure of the primary affections will not secure patients from the subsequent eruption of secondary syphilis, or in the still stronger language of M. Desruelles, "l'usage du mercure a la plus grand part dans la production des symptômes secondaires," they say that it is only applicable to its imperfect employment: confident in their principle that mercury, systematically administered during the primary stage of the complaint, will prevent its further development, they look upon the occurrence of secondary symptoms as a proof of the inadequacy of the treatment of the primary affection, and as somewhat discreditable to the practitioner who conducted it. Nevertheless, they do not adhere to this principle as absolute; since constitutional affections, under certain circumstances, will follow in despite of any method of treatment: but these are sufficiently rare, say they, to authorize us to regard them as exceptions to the rule, and not as subversive of it. What this systematic exhibition of mercury consists in, we shall now proceed to enquire; and as we look upon the observations of Dr. Colles as the most precise and practical that have yet appeared on the subject, we shall turn to his work for our chief materials.

At the same time that he dissents from the rigid discipline insisted on by our ancestors during a mercurial course, Dr. C., contrary to the opinion of Hunter, and coinciding thus far with the views of the partisans of the simple treatment, urges the necessity of some measures preparatory to the exhibition of mercury in syphilis, believing that much less mischief was produced by the old system than that which accrues from the laxity of modern practitioners in this respect. "Not only may this powerful medicine disappoint our expectations and occasion serious evils when its use is commenced under certain morbid conditions of the system, but the same may happen in the young and healthy if they are hurried into a mercurial course without any preliminary attention to their condition, or without any particular instructions as to their mode of living." Dr. Colles does not specify the measures which he considers necessary, but we may gather from various parts of his work, that these consist chiefly of moderation in diet and regular habits of life, with strict attention to the proper action of the secretory and excretory functions.

Before entering into the particulars of his mercurial treatment, Dr. C. offers a few observations on the curative action of mercury, which he believes to be almost always coincident with its affection of the salivary organs; "it is surely, therefore, (he says,) a fair and legitimate conclusion to affirm that ptyalism marks the salutary operation of this mineral." He is anxious, nevertheless, that it should not be imagined, from the foregoing statement, that he is disposed to measure the efficacy of mercury by the amount of salivation which it excites. A slight increase of secretion, accompanied by swelling and superficial ulceration of the gums, is all that he aims at effecting; and this he regards as indicating, first, that the mercury is acting in a salutary manner upon the system; and, secondly, that it displays a degree of power which will suffice to eradicate the disease.

Dr. Colles, then, considers moderate ptyalism as essential to and the test of the beneficial operation of mercury. A timid use of the medicine, that is to say, either a great diminution in quantity, or an absolute abandonment as soon as salivation comes on, (at which time a chancre will frequently put on a more unfavorable aspect,) is worse, both in primary and secondary syphilis, than proscribing its use altogether; as, by this proceeding, the health becomes disturbed, and the local disease takes on a stubborn character, which is not soon removed; and, where secondary disease exists, the symptoms become masked.

"The true rule by which we should guide our practice is this: to desist from the further use of mercury in the advanced stage of a mercurial course, as soon as we perceive a decided change for the worse in the ulcers (or rather symptoms). Let us not confound together the bad changes which take place in the onset of mercurial treatment before this medicine has taken full hold of the system, with those bad changes which we occasionally witness in the advanced stage of the course, when the mercury has for some time had full power over the system." (P. 172.)

When mercury does not produce salivation at, or soon after, the usual period, but its effects are confined to ulceration of the gums and fever, the "dry course" of the lower orders in Ireland, Dr. Colles has often succeeded by reducing the doses and lengthening the interval between them. In whatever form the remedy is used, the general rule attempted to be followed is, to induce ptyalism, without any severe constitutional disturbance, within from seven to ten days. If salivation is suddenly excited, the curative action of mercury is not to be relied on; the primary disease may be removed by it, "but secondary symptoms will not fail to make their appearance in full vigour. After such sudden and profuse salivations, (adds Dr. C.) I have uniformly found the symptoms very unmanageable, and by no means yielding in the usual way to a subsequent use of mercury." The first six or eight days, therefore, being the most critical period of the course, during that time great caution must be enjoined, as he is convinced that many of the failures in the mercurial management of syphilis arise from inattention to this important rule. When once the system has been brought fairly under the influence of mercury, there is then little to fear from its protracted use, provided it is not allowed to pass beyond the line already pointed out, and the state of the general health is not neglected.

Dr. Colles gives a clear account of the various idiosyncrasies observed in regard to the action of mercury, and points out the different morbid affections produced by it. One of the most unerring proofs that the medicine is too powerful in its operation relatively to the constitution of the individual, is the occurrence of excoriation round the anus. Mercurial erythema is only to be apprehended at that period of the course when ptyalism is *first* excited. It must, therefore, be a rule to watch for it then, and to discontinue the mercury instantly it appears. There is no danger of this affection at any subsequent period. Mercurial erethismus, on the contrary, only arises late in a course, and generally comes on when an increased dose has been given to excite salivation, or when that has been allowed to subside and we are endeavouring to reproduce it. The author is perfectly confident that it will not attack any person in a state of moderate ptyalism.

Dr. Colles's treatment of the individual forms of syphilis is marked by his usual practical good sense. His opinion that the rapid healing of primary sores under stimulants is no security against consecutive affections, has been already noticed. In the management of the true Hunterian chancre, the local applications ought to be of the blandest nature, so as not to alter the natural features of the ulcer; from observing which, he is convinced that so many useful indications to guide us in the administration of mercury, may be drawn. On the other hand, he believes that the occurrence of secondary symptoms is not prevented by the early application of caustics, or even by cutting out the chancre, of the failure of which he has seen very decided instances. He does not agree with Hunter in the opinion that the circulation may be long loaded with mercury before the chancre becomes affected by it. Ordinarily the constitution is brought under its influence between the third and eighth day; and he feels no hesitation in saying, that as soon as this takes place, we shall observe a striking change in the appearance of the ulcer. The first remarkable change is, that the chancre appears a little larger, but, at the same time, less deep; we next find that the surrounding hardness declines, that granulations begin to arise, that the discharge becomes purulent, and that the entire surface of the ulcer becomes clean and red. In a few days more, the ulcer contracts, and a thin cuticle forms on its edges, and this daily increases until it is finally healed. Some degree of hardness, however, remains for several days after it has healed, but this also disappears and leaves the part possessed of its natural softness.

"I shall only add, (pursues Dr. C.) that, in general, it will be prudent to continue the use of mercury not only until all hardness be removed, but even for a few days longer. I think we may lay it down as a rule, that the course of mercury, even when it has been well conducted and has agreed with the patient, should be continued, to the extent of moderate ptyalism, for not less than one month."

The advice given by Mr. Judd on this point is the same.

"If mercury be given for the removal of primary contamination by venereal virus, the use of that mineral should be continued from twenty-four to thirty days after the gums become affected by the remedy; and an adequate quantity should be taken to keep up a peculiar coppery taste in the mouth, especially on waking in the morning, and also to cause a slight but steadily continued tenderness and redness of the gums, perceptible to the eye on inspecting that portion supporting the incisores teeth. If a less quantity than that be administered, the patient will run all the risk of injury, without deriving the benefit of security: on the contrary, absorption will have been promoted, and in many instances cause the secondary symptoms—those direful affections which we are called upon either to prevent or remove." (P. 555.)

We shall not follow Dr. Colles through his details relating to the treatment of secondary syphilis under ordinary circumstances. The same principles apply to this as to the primary affection, with the distinction that in this form the action of the medicine will usually require to be kept up, in ulcerations of the throat especially, for eight or ten weeks. His observations on inveterate syphilis, relapsed sore throat, pains in the limbs, nodes, and venereal swelled testicle, must not be passed over so cursorily. We have before referred to his opinion that the injudicious use of mercury, or the misconduct of the patient during its exhibition, has, still, a large share in inducing some of these pernicious consequences and in rendering many of them most unmanageable. These cases were a stumbling-block to the old mercurialists; and it is no wonder that the terrible consequences resulting from their persistance in an unmeasured use of mercury, should have led to its recent proscription. The practice of Dr. Colles differs very little from that of the moderate adherents of the new doctrine, who, whilst they deny the claims of mercury to the character of a specific in syphilis, turn to it as the last and only resource in inveterate secondary affections. He employs it in such cases, in smaller doses and with greater precautions than under ordinary circumstances, combining with it means for the improvement and support of the general health; and he finds his principle of treatment, namely, gentle ptyalism steadily kept up, here, also, completely borne out by success. In venereal hectic fever, which has hitherto been looked upon as a state peremptorily forbidding the use of mercury, Dr. C. is convinced that it may be administered not only with safety but a certain expectation of effecting a cure. He begins it, under these circumstances without preparation, in minute doses, prescribing Pilul. Hydrarg. gr. iij. with Extract. Cicutæ gr. ij., to be taken once or twice a day, combined with sarsaparilla.

In extreme cases, Dr. C. disregards the two maxims not to give mercury during febrile excitement nor whilst an eruption is coming out; but the state of eruptive fever, as well as that of hectic and exhaustion, require that the mercury should be employed in a manner peculiarly suited to such conditions.

"Were we to use mercury with these, as we do with venereal patients in general, I believe we should commit most serious mischief. In these cases we should not commence with a larger dose than ten grains of ungt. hydr. fort. every morning, or with an equivalent of blue pill; that is, about gr. iij. mane nocteque. However inconsiderable such doses may appear, still we shall be gratified to find that on the third or fourth day the mercury is acting in the most favorable and salutary manner on the system; and it but seldom occurs that these effects are deferred to the sixth or seventh day." (P. 234.)

In his treatment of syphilis in scrofulous patients, Dr. C. has no dread of the injurious effects of mercury; he deals with this complication exactly as if scrofula were not present, with this distinction that he is anxious to excite a smart ptyalism with the least possible delay.

The general principle on which Dr. Colles conducts the mercurial treatment of syphilis, is amply supported by practical experience both in this country and on the continent. In Germany, Louvrier, Rust, Wedemeyer, Simon, &c. maintain corresponding views; and the minutes of the meeting at Nantes contain a respectable mass of evidence in its favour derived from our French brethren. A large majority of the speakers declared themselves for the mild mercurial treatment in both primary and secondary disease, at the same time recognizing the value of a previous and contemporaneous employment of antiphlogistic measures.

In the treatment of syphilitic diseases, then, the surgeon has the choice of two lines of conduct. He may, in the first place, endeavour to cure the local malady by the simple method, to accomplish which no better rules can be suggested than those laid down by M. Desruelles, although they form a tolerably severe system of discipline; and if, on the failure of this attempt, or the subsequent occurrence of consecutive symptoms, the specific nature of the disease is more fully displayed, he can then recur to the specific remedy.

Or, secondly, he may give mercury soon after the outbreak of the primary disease; not, however, neglecting hygienic measures; and continue it until he is satisfied that all danger of consecutive disease is at an end; by this proceeding he will be enabled, except under circumstances of rare occurrence, confidently to assure his patient that no ulterior consequences will trouble him; but, at the same time, he will have treated by a process tedious, disagreeable, and sometimes injurious, a large proportion of cases which would have rapidly got well under ordinary measures.

IV. It only now remains to examine briefly the merits of the several works at the head of the article, and the share which they have respectively had in supplying the materials for its composition.

1. M. Desruelles, the author of the first work on our list, has long been distinguished as a zealous disciple of Broussais. After serving as a regimental surgeon in the French army for some years, he was appointed to

4282

the charge of the venereal wards of the Hospital of the Val de Grace in the year 1825. In 1827, he published a volume of memoirs on the treatment of venereal maladies without mercury, and in 1828 and 1829, contributed papers "on the comparative results obtained by the mercurial and non-mercurial methods of treatment employed at the military hospital of the Val de Grace," to the 25th and 27th vols. of Mem. de Médecine et de Chirurgie Militaires. The work before us contains the results of his experience from August 1827, to April 1835; during which period he treated 8810 cases of venereal disease. It is divided into two parts; the first contains a general view of the history, theories and treatments of venereal maladies; the second, a description of them in detail, with indications of the suitable modes of treatment according to the principles of the new doctrine. To the end of the work is appended a formulary of the various medicinal substances which still maintain a reputation in the treatment of these diseases; they are arranged in alphabetical order, and, under each remedy will be found the most approved formulæ for its exhibition. Nor must we pass unnoticed a copious bibliography at the commencement, in which the pages of the treatise, where any particular author is referred to, are indicated.

An elaborate sketch of the history of syphilis occupies the first 147 pages. This is, in fact, almost exclusively, a report on the rise and progress of the non-mercurial treatment, and is, on that ground, well deserving attention. Many of the early facts on the comparative merits of the mercurial and non-mercurial systems of treatment are derived from British sources, the beneficial influence exercised by them on our practical views having been already shown by Mr. Bacot in his very perspicuous monograph of these maladies. But the additional experiments, some of them conducted on so large a scale, which have been industriously collected by M. D., furnish us with much more extended and valuable data on which to found an opinion.

Any lengthened discussion of the author's too ingenious and accommodating theory, a mere expansion, as he avows, of the principles of Broussais, would lead us into a critique on the doctrine of the latter. Were it not that M. Desruelles deviates in practice from his theoretical dogmas (in which he is by no means singular among writers on syphilis,) we should be tempted to apply to him and to his coadjutors the words of M. Cruveilhier, in his inaugural discourse for 1836, "Ce sont les métaphysiciens ou les pöetes de la science, ils ne seront jamais praticiens."

The ease with which every phenomenon of the disease is accounted for by this theory ought to have raised a suspicion of its validity; and, doubtless, some such misgiving did cross the author's mind when he thought it advisable to state apologetically that he "attached comparatively little importance to it; but that he had traced out some hypothetical views, because it was necessary to harmonize the facts of the ancient doctrine with those of the new."

In that portion of the work entitled a consideration of "some questions relative to venereal maladies," a great mass of information on their propagation, characters, progress, and termination are brought together, showing how much has been effected within the last few years towards illustrating the natural history of the disease.

The second part of the treatise, also, contains much valuable matter.

The mercurial treatment on which he relies when simple measures are unsuccessful, both in the preparations employed and the manner of exhibiting them, scarcely differs from that followed by many sound practitioners in this country. There is, however, this material distinction between them,—that M. D. cures as many local or primary diseases as he can by the same means he would resort to in accidents where no venereal origin is suspected, reserving the more potent remedy for obstinate primary, and for consecutive affections, which latter he looks upon as extreme cases under this proceeding.

Regarded as a work of science, its great blemishes are the hypothetical views, above alluded to, and its partiality. Everywhere the author betrays an undue feeling of partisanship. An aggravated picture of the evils of the mercurial treatment is continually placed before the eye, in order to enhance the merits of the simple treatment. In his reports and examination of facts drawn from the history of the disease, impelled, no doubt, by his anxiety to "harmonize" those of the old and new doctrines, his appreciation of the evidence, and of the parties from whom it is derived, is not always quite honest. Whenever the testimony is favorable to his system, the writer is introduced to us as a "physiologiste profond," "praticien distingué," "médecin experimenté," &c. On the other hand, those who furnish evidence which does not accord with the author's principles are sometimes stigmatized, most gratuitously, as unworthy of belief.

These drawbacks notwithstanding, M. Desruelles has displayed a talent and research in conducting his enquiry into the nature and treatment of a disease involved in so much perplexity, which entitle him to high praise.

2. As the treatise of M. Desruelles may be designated a manifesto of the new non-specific doctrines, so may that of Dr. Colles be fairly considered as embodying the opinions, theoretical and practical, of that large and, we venture to affirm, increasing class of practitioners, who still hold that syphilis is a disease of a specific nature, and that mercury is the only remedy on whose curative power we can safely and confidently rely. It is not to be compared with the work of Mr. Bacot, as a clear and compendious digest of the entire subject; in fact, that has not been the author's aim. He has scarcely alluded to the labours of others, but lays before us a simple statement of his own views, drawn from a long and extensive experience in the management of the disease, together with certain theoretical opinions naturally suggested to his mind by an attentive observation of its various phases.

We have given so prominent a station to Dr. Colles's opinions upon all the most important parts of our subject, that we might fairly leave the reader to form his own judgment of the merits of the work; but we feel that we have not been able, within limits so narrow, to do justice to its great practical value. In the present vacillating state of opinion on the treatment of the disease, in which neither system has fair play allowed it, we must rank as a most important accession to our knowledge, Dr. Colles's demonstration of the different effects produced by different modes of administering mercury; its beneficial application to varieties of inveterate syphilis, which assumed so destructive a character in the hands of practitioners of the old school, and so frequently baffle the efforts of disciples of the new doctrine, if fully confirmed, will restore mercury to its former high station, from which an indiscriminating routine practice has deposed it. The author may, possibly, feel too sanguine with respect to the general success which he anticipates from his modified treatment, but the practice of the most experienced surgeons of the present day goes far to corroborate his statements; and it is worth noting, that those who dispute the value of the system, advance no satisfactory method of treatment as a substitute for it. We therefore strongly recommend Dr. Colles's observations to the consideration of practitioners of every shade of opinion, confident that they will rise from the perusal, if not with a clearer insight into the nature of the disease, at any rate with more fixed principles for treating it.

3. *The Minutes of the Meeting at Nantes* is a thin volume of some 150 pages, which has made its appearance at a highly seasonable juncture. It is interesting as a summary, to a certain extent, of the state of opinion regarding the two methods of treating syphilis, and as a proof of the jealous scrutiny to which the new doctrine is submitted by our neighbours on the other side of the Channel.

In March, 1835, a proposition was made by the members of the medical section of the Royal Academic Society of Nantes, to those societies with which it was in correspondence, that a discussion should take place on the two questions of the existence or non-existence of a syphilitic virus, and the comparative advantages of the mercurial and non-mercurial plans of treatment. At the same time, the practitioners of Nantes were invited to assemble, and organize a regular series of meetings, at which the opinions of each individual might be made known and collected. On the 2d of July, 1835, the first meeting was held in the hall of the Hôtel de Ville of Nantes; upwards of fifty physicians of that town and its vicinity being present. Among them M. Devergie, author of the "Clinique de la Maladie Syphilitique," took his place, and was charged by M. Desruelles to express his regret that he could not also be present.

The discussion continued during four meetings, and was conducted in a manner highly creditable to the ability of the parties engaged in it. How great soever the differences with regard to theory may have been, as far as practice is concerned, the average opinion expressed indicates a blending together of the old and new doctrines to a greater or less extent; the principles and practice of the former have been favorably modified by those of the latter; but, in the extreme views for which they contended, M. Devergie and his companions found the strength of the meeting, both in number and argument, arrayed against them.

4. The work of Dr. Oesterlen is an enlarged edition of an Essay on the Unity or Plurality of the Venereal Contagion, to which a prize, offered in 1833, by the Faculty of Tübingen, for the best essay on that subject, was adjudged. It is divided into two parts; the first contains an examination of the question as to the identity or difference of the gonorrhœal and chancrous contagions; the second takes a general survey of the various disputed points with regard to the identity or difference of the syphilitic and the so-called pseudo-syphilitic contagions. In the prosecution of this enquiry, Dr. Oesterlen has exhibited great research and a considerable share of discrimination. He has brought together English, American, French, and German authorities, and contrasted them with impartiality and acuteness. We have availed ourselves of the materials collected by him, whenever they had a marked practical bearing, but have carefully avoided plunging into the intricacies of this question, which, after all his labour, he confesses his incompetency to decide. Those who desire to enter more fully into the numerous questions springing from this discussion will nowhere find a more clear and comprehensive display of them.

5. The work of Dr. Dieterich is rich in lore, but, like too many of the productions of the German press, is verbose and diffuse. It contains, however, so much valuable information on the subject to which it is devoted, that we may perhaps return to it on another occasion.

6. The excellent work of Dr. Oppenheim contains many judicious views and much important knowledge, conveyed in a concise form. The comparatively old date of the publication would render any lengthened account of it in this place improper; but we recommend it to the surgical student as a valuable document.

7. In the work before us, M. Baron Boyer* sets out with the assertion that the diagnostic mark between syphilitic primary affections and similar diseases arising from ordinary causes, is the coexistence of a bubo with the former. "The development of a bubo, where primary disease exists, however small it may be, is not to be attributed to sympathy, but to the syphilitic infection; and the coincidence of bubo with inflammation of the urethra and vagina, or with an ulcer, proves that the individual has contracted syphilis." Although M. Boyer adopted this opinion three years back, he candidly acknowledges that he has not hitherto succeeded in convincing those of his friends to whom he has communicated his ideas. We doubt if he will be more successful in his endeavours to convert others. General experience militates so strongly against such a notion, that it is not worth while to attempt a formal refutation of it. Into the author's classification of primary and secondary syphilis we cannot enter. It is plain that he has laid his peculiar opinions too hastily before the public to have been able to test them fairly by experience. His matter, therefore, has not quite value sufficient to compensate for the rather dogmatic manner which he has generally adopted. He has added little or nothing to our knowledge of the theory or treatment of this class of diseases.

8. If the work of Mr. Judd is to be received by our continental brethren as a specimen of the manner in which the complicated questions regarding syphilis are treated in this country,—as our contribution, in 1836, towards their elucidation,—it will not, we fear, tend to remove the imputation cast by M. Desruelles on the qualifications of British practitioners. The very title-page is calculated to produce, at the outset, an impression unfavorable to the author, which, we are sorry to think, other parts of the work serve rather to increase. Some peculiarities in the manner in which he lays his information before his readers we ourselves ascribe purely to a want of good taste; but he must not be surprised if they excite, in the less charitably disposed, a suspicion that he is more

* Son of the late celebrated chief surgeon to the hospital of la Charité.

desirous of winning golden opinions of that "indulgent public," to whose decision he appeals in the preface, than simply of "contributing his mite towards the general good." The volume consists of two parts; the first embraces urethritis, and its consequences; the second is devoted to syphilis, and is preceded by a series of anatomical and pathological observations on the "dermoid coverings," prefatory to his classification of syphilitic cutaneous affections. We have elsewhere glanced at Mr. Judd's theoretical views, as well as his classification of secondary diseases, and noticed his "proposal of a substitute for mercury." With the exception of a few very meager observations at the end of the volume, the reader is left to glean the author's method of treatment from his tediously detailed cases, which occupy little less than three-fourths of the whole work. His chief practical deductions are, that small quantities of mercury, employed for a short period, instead of curing the disease, lead rather to contamination; and that ptyalism is not always a specific. Mr. Judd's language is sometimes so perplexed as to be with difficulty intelligible; a defect which appears to arise as often from confusion of ideas as from obscurity of style merely dependent on a want of familiarity with his pen.

9. Of the *Treatise of the Venereal Disease*, by Hunter, it is unnecessary to say much, as both its excellencies and defects have been long properly appreciated by the profession in this and other countries. Although, as Mr. Babington remarks, "tainted, more than any of the other works of John Hunter, with the vice of a too-hasty generalization, yet it is full of practical observations of the highest value, and must always form an essential part of the study of every surgeon who wishes to make himself acquainted with the disease of which it treats."

Under the hands of Mr. Babington, who has performed his task as editor in a very exemplary manner, the work has assumed quite a new value, and may now be as advantageously placed in the library of the student as in that of the experienced surgeon. We cannot characterize the improvements in the new edition with more accuracy and truth than in the concluding words of the editor's preface. "It has been attempted to render it more generally useful, by ingrafting on it the labours of other surgeons. The opinions of the author have been treated with the respect which is due to his high reputation; but, where important facts have been brought to light by others, either in confirmation or in correction of his principles, they have been presented to the reader, in order that he may have before him in one view the present state of the science, and that, by comparing the reasoning of others with that of the author, he may be enabled to form a just opinion of the truth or the error of his conclusions." It may account for the slight use we have made of Mr. Babington's notes in the present article, to state that the work, although only published in the present year, was really printed in the year 1835.

